# Reconsidering evidence for psychedelic-induced psychosis: An overview of reviews, a systematic review, and meta-analysis of human studies

**DOI:** 10.1192/j.eurpsy.2025.710

**Published:** 2025-08-26

**Authors:** M. Sabé

**Affiliations:** 1 Adult Psychiatry, Geneva University Hospitals; 2 Faculty of Medicine, University of Geneva, Geneva, Switzerland

## Abstract

**Introduction:**

Persons with schizophrenia are currently excluded from psychedelic-assisted therapy due to concerns about psychedelic-induced acute or persistent psychotic symptoms. However, meta-analytic evidence of the precise risk for psychedelic-induced de novo and exacerbation of psychosis in people with pre-existing psychotic disorders is lacking.

**Objectives:**

We conducted an overview of reviews, systematic review, and meta-analysis to examine the incidence of psychedelic-induced psychosis and the exacerbation of psychotic symptoms in schizophrenia.

**Methods:**

Our pre-registered protocol (CRD42023399591) covered: LSD, psilocybin, mescaline, DMT, and MDMA. Embase, PubMed, PsyARTICLES, PsyINFO, and trial registries were searched from inception until 11/2023.

**Results:**

The incidence of psychedelic-induced psychosis was computed using a random-effects model, and standardized assessments of study quality was performed. We retained 131 publications: 14 systematic reviews, 20 reviews, 35 randomized-controlled trials (RCTs), 10 case-control studies, 30 uncontrolled trials (UCT), and 22 cohort studies with overall low study quality. The meta-analysis included nine studies. Incidence of psychedelic-induced psychosis was 0.002% (95%CI 0-0.006, I^2^=0%, N=123,800; n=2) in population studies; 0.2% (95%CI 0.1-0.3, I^2^=0%, N=6,535; n=6) in UCT, and 0.6% (95%CI 0.2-1.8, I^2^=0%, N=563; n=3) in RCTs excluding individuals with a history of psychotic symptoms. In UCT including patients with schizophrenia, 3.8% (95%CI 1.6-8.9, I^2^=0%, N=133; n=2) developed long-lasting psychotic symptoms. In cohort studies, 13.1% (95%CI 9.4-17.9, I^2^=24%, N=353; n=3) of those with psychedelic-induced psychosis developed schizophrenia. Sensitivity analyses confirmed the main findings. The incidence for psychedelic-induced psychosis is low but slightly higher in studies including patients with schizophrenia. The risk of transition to schizophrenia after psychedelic-induced psychosis is considerable.

**Conclusions:**

In summary, the reviewed evidence suggests that schizophrenia might not be a definite exclusion criterion for clinical trials exploring safety and efficacy of psychedelics for treatment-resistant depression and negative symptoms. However, given the low quality and limited number of studies, more high-quality research is needed, and a conservative approach is recommended until further data is available.

**Disclosure of Interest:**

None Declared

